# The Role of the RACK1 Ortholog Cpc2p in Modulating Pheromone-Induced Cell Cycle Arrest in Fission Yeast

**DOI:** 10.1371/journal.pone.0065927

**Published:** 2013-07-03

**Authors:** Magdalena Mos, Manuel A. Esparza-Franco, Emma L. Godfrey, Kathryn Richardson, John Davey, Graham Ladds

**Affiliations:** 1 Division of Biomedical Cell Biology, Warwick Medical School, University of Warwick, Coventry, United Kingdom; 2 Systems Biology Doctoral Training Centre, University of Warwick, Coventry, United Kingdom; Cancer Research UK London Research Institute, United Kingdom

## Abstract

The detection and amplification of extracellular signals requires the involvement of multiple protein components. In mammalian cells the receptor of activated C kinase (RACK1) is an important scaffolding protein for signal transduction networks. Further, it also performs a critical function in regulating the cell cycle by modulating the G_1_/S transition. Many eukaryotic cells express RACK1 orthologs, with one example being Cpc2p in the fission yeast *Schizosaccharomyces pombe*. In contrast to RACK1, Cpc2p has been described to positively regulate, at the ribosomal level, cells entry into M phase. In addition, Cpc2p controls the stress response pathways through an interaction with Msa2p, and sexual development by modulating Ran1p/Pat1p. Here we describe investigations into the role, which Cpc2p performs in controlling the G protein-mediated mating response pathway. Despite structural similarity to Gβ-like subunits, Cpc2p appears not to function at the G protein level. However, upon pheromone stimulation, cells overexpressing Cpc2p display substantial cell morphology defects, disorientation of septum formation and a significantly protracted G_1_ arrest. Cpc2p has the potential to function at multiple positions within the pheromone response pathway. We provide a mechanistic interpretation of this novel data by linking Cpc2p function, during the mating response, with its previous described interactions with Ran1p/Pat1p. We suggest that overexpressing Cpc2p prolongs the stimulated state of pheromone-induced cells by increasing *ste11* gene expression. These data indicate that Cpc2p regulates the pheromone-induced cell cycle arrest in fission yeast by delaying cells entry into S phase.

## Introduction

Eukaryotic cells are constantly exposed to different stimuli and are therefore required to both interpret and integrate their response to these signals in order to modulate their behavior. Many external signals are detected through cell surface G protein-coupled receptors (GPCRs), which couples to heterotrimeric G proteins consisting of a Gα, Gβ and a Gγ. In the inactive state, a Gα subunit is bound to a molecule of GDP. Upon agonist stimulation of a GPCR, nucleotide exchange occurs upon the Gα subunit such that GDP is lost and replaced by GTP. This promotes disassociation of Gα-GTP from the Gβγ dimer. Each can then regulate the activity of effector proteins thereby bringing about changes in cellular behavior [1]. Signaling is terminated when Gα-GTP is hydrolyzed to GDP through the intrinsic GTPase activity of the Gα subunit leading to the re-association of the heterotrimer.

The Gβγ-dimer can function at different levels to regulate G protein signaling. Most Gβγ-dimers recruit Gα-subunits to the plasma membrane facilitating interactions with agonist-bound receptors. However, they can also act as guanine nucleotide disassociation inhibitors (GDIs) by blocking the spontaneous exchange of GTP for GDP on the Gα subunit. Finally, Gβα-subunits can act as signal transducers within their own right by activating proteins such as adenylate cyclases and specific G protein-inward rectifying potassium channels [2]. A number of specific Gβγ-modulating/activating proteins have been identified including the activator of G protein signaling (AGS) superfamily [3]–[5].

In recent times it has become evident that G protein-mediated signaling cascades do not always require classical Gβγ-subunits. One such example is the glucose-sensing pathway in the budding yeast *Saccharomyces cerevisiae* where a number of Gβ-structural mimics have been reported. These include two kelch-repeat containing proteins Krh1p/Gpb1p and Krh2p/Gpb1p (however these proteins are now known to act further downstream of the Gα subunit [6]–[9]) and more recently a WD-repeat protein, Asc1p [10] an ortholog of mammalian receptor of activated protein C kinase (RACK1). It has been speculated that Gα subunits in other GPCR-mediate systems may interact with non-classical Gβγ-like proteins, and one such example is the pheromone-response pathway of fission yeast [11].

During the mating response *Schizosaccharomyces pombe* cells exchange pheromones that bind to cell surface GPCRs [12], and transduce their signals via Gpa1p (Gα subunit) through a classical mitogen-activated protein (MAP) kinase cascade, resulting in activation of the transcription factor Ste11p. Critical for efficient mating of the cells, is their resultant entry into a transient G_1_ arrest following pheromone stimulation [13]. Based upon sequence and structural comparison of typical G protein subunits within the *S. pombe* genome, there appears to be only one canonical Gβγ-subunit (Git5p/Git11p). Early research suggested that Git5p/Git11p could function on the pheromone cascade [14], however this now appears to be incorrect [15]. We have recently reported, through the use of a yeast 2–hybrid screen to identify interacting partners of Gpa1p, the isolation of a Gβ-like subunit, Gnr1p [16]. Both disruption and overexpression of Gnr1p demonstrated its role as a negative regulator of Gpa1p but it was not required for signaling [16]. As part of the same screen, we identified a second weak interacting partner of Gpa1p, Cpc2p.

Cpc2p is a Trp-Asp (WD)-repeat protein, and is the *S. pombe* ortholog of both RACK1 and Asc1p [17], [18]. RACK1 is highly conserved among eukaryotic species [18], [19], and it was originally described for its ability to interact with specific protein kinase C isoforms. In addition, it has become apparent that RACK1 is involved in complex cellular signal transduction pathways and chromatin organization [18], [19]. Further, it also plays a role in mitotically growing cells by delaying entry into S phase [20], [21].

Analogous to RACK1, Cpc2p appears to regulate a wide range of responses within *S. pombe*. It has also been suggested that Cpc2p modulates sexual development though its action on Ran1p/Pat1p, so regulating the transition from mitosis to meiosis [17], [22]. In addition, Cpc2p controls the stress response pathway through regulation of Atf1p via an interaction with Msa2p [18] although the precise nature of this modulation remains unknown. It has however, been demonstrated that deletion of *cpc2* is epistatic to an *msa2* deletion suggesting that Msa2p may negatively regulate Cpc2p [22]. Further, Msa2p performs a number of cellular roles including modulating the stability of *cdc4* mRNA, (Cdc4p encodes an essential light chain of myosin and plays a crucial role in cytokinesis) [23], and regulates the onset of sexual differentiation by repressing Ste11p-regulated genes [24]. The interplay between Msa2p and Ste11p regulated genes appears complex since, Msa2p is itself, negatively regulated by pheromone. Upon pheromone-stimulation activated Spk1p (the MAP kinase of the mating response) reduces Msa2p activity, allowing an increase in Ste11p translation [25]. Cpc2p appears to have a number of other cellular roles including; modulating the cell cycle of mitotically growing cells by regulating the G_2_/M transition [26] and a documented association with the 40S ribosomal subunit [27], that suggests a role in modulating the level of translation for many other genes.

Here we describe a novel and distinct role for Cpc2p within the *S. pombe* pheromone-response pathway. Despite initially isolating Cpc2p as a structural Gβ-mimic for Gpa1p, we now suggest that, Cpc2p also performs an important role in modulating *S. pombe* cells entry into S phase following pheromone-induced cell cycle arrest. We attempt to provide a molecular explanation for this data by linking Cpc2p modulation of Ran1p/Pat1p to pheromone signaling and also highlight a potential role for Msa2p in this process.

## Results

Sequence and structural comparisons of typical G protein subunits within the fission yeast genome have identified a single canonical Gβγ (Git5p/Git11p). Evidence has been presented suggesting that Git5p and Git11p do not function on the pheromone-response pathway but solely act to module Gpa2p from the glucose-sensing pathway [15]. In a previous study, we described our use of the yeast-2 hybrid system to identify Gpa1p-binding partners [16]. From this screen we isolated a number of interactants, including the WD-repeat protein, Gnr1p. Quantitative characterization (Figure S1) [16] using previously described reporter strains (JY546; h^−^, *cyr1* ^−^, *sxa2>lacZ*) where the bacterial enzyme β-galactosidase transcription is linked to the promoter of the Ste11p-regulated pheromone-responsive gene *sxa2* (Sxa2p is a carboxypeptidase that is only expressed after pheromone stimulation [28], [29]) confirmed Gnr1p as a negative modulator of the pheromone-response pathway in fission yeast.

**Figure 1:**
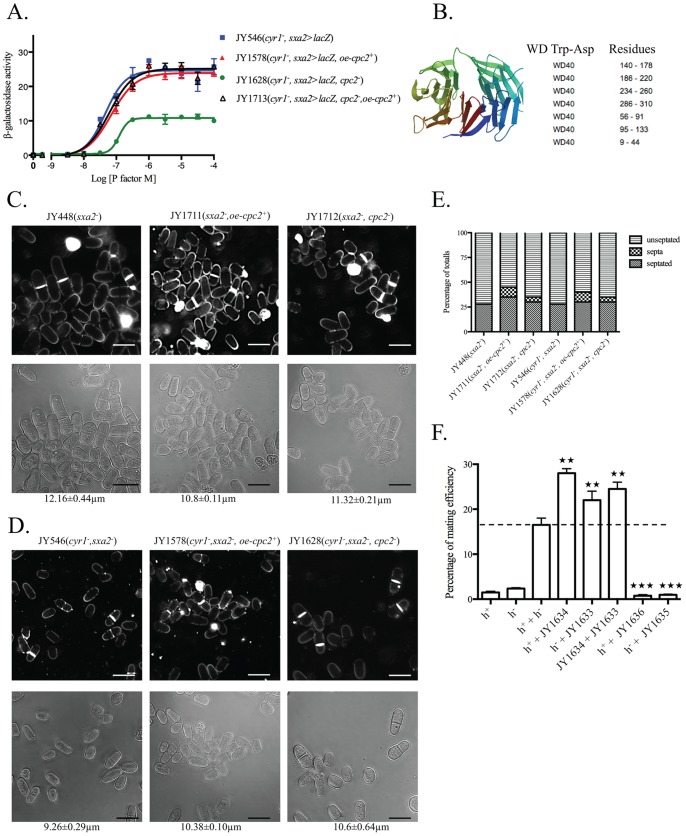
Characterization of potential Gβ-subunit mimic in the pheromone-response pathway. (A) Pheromone-dependent transcription for the strains JY546 (h^−^, cyr1^−^, sxa2>lacZ), JY1578 (h^−^, cyr1^−^, sxa2>lacZ+oe-cpc2^+^), JY1628 (h^−^, cyr1^−^, sxa2>lacZ, cpc2^−^) and JY1713 (h^−^, cyr1^−^, sxa2>lacZ, cpc2^−^+oe-cpc2^+^) was determined using the sxa2>lacZ reporter. Values are means of triplicate determinations ± S.E.M. (B) Molecular structure as determined using the SWISS-MODEL Repository for Cpc2p. Cpc2p contains seven WD-40 repeat domains with positions of residues as indicated by colors on the molecular structure. (C) Cell morphology and size, at division (micrometers ± S.D.) for strains JY448 (h^−^, cyr1^−^, sxa2^−^), JY1711 (h^−^, sxa2^−^+oe-cpc2^+^), JY1712 (h^−^, sxa2^−^, cpc2^−^), grown in minimal medium at 29°C and stained with calcofluor white (top panel) or imaged through using bright field microscopy on agar pads (bottom panel). Scale bars 10 µm. (D) As for C except strains JY546 (h^−^, cyr1^−^, sxa2>lacZ), JY1578 (h^−^, cyr1^−^, sxa2>lacZ+oe-cpc2^+^), and JY1628 (h^−^, cyr1^−^, sxa2>lacZ, cpc2^−^) were used. (E) Numbers of non-septated, septated and multiple septa containing cells for the strains in C and D were determined from 400 individual cells. Values shown correspond to the percentages of the total population. Cells were stained with calcofluor white, to enable visualization of septum material. (F) Mating efficiency was quantitated for cells overexpressing (JY1634, h^−^, +oe-cpc2^+^; JY1633 h^+^, +oe-cpc2^+^) or lacking Cpc2p (JY1636, h^−^, cpc2^−^; JY1635 h^+^, cpc2^−^) compared to M (JY402, h^−^) and P (JY383, h^+^) control cells. Statistical significance determined compared to M (h^−^) and P (h^+^) using a one-way Anova with a tukey multiple comparison post test where ★★★ represents p≤0.001, ★★ represents p≤0.01, and ★ represents p≤0.1.

A second Gpa1p-binding partner identified in our yeast-2 hybrid screen [16], but not extensively characterized was Cpc2p. Deletion of the *cpc2* ORF from our Ste11p-regulated pheromone-responsive *sxa2>lacZ* reporter strains (JY1628; h^−^, *cyr1* ^−^, *sxa2>lacZ, cpc2* ^−^) resulted in cells displaying a reduced sensitivity to pheromone (Wild type (JY546) - pEC_50_ = 7.3±0.07; *cpc2* ^−^ (JY1628) - pEC_50_ = 6.9±0.03) and a two-fold decrease in the extent of the maximal response (Wild type (JY546) - E_max_  = 24.45±0.73 units; *cpc2* ^−^ (JY1628) - E_max_  = 10.82±0.22 units) (Figure 1A). These defects were rescued upon expression of Cpc2p from an inducible plasmid. Overexpression of Cpc2p in cells containing an endogenous copy of *cpc2*, did not significantly alter their sensitivity or maximal response to pheromone. These results are in contrast to that observed for Gnr1p (Figure S1), and suggest that Cpc2p is not a negative modulator of pheromone signaling, despite its potential interaction with Gpa1p. Attempts to validate a potential *in vivo* interaction between Gpa1p and Cpc2p using co-immunoprecipitation have proven to be unsuccessfully (data not shown) and indicate a potential weak interaction between these two proteins. Subsequent mutational analysis of the N-terminal 40 amino acids of Gpa1p suggested that these residues alone are necessary and sufficient to enable Gpa1p association with plasma membrane structures in the absence of either Cpc2p or Gnr1p (Figure S2). These results would suggest that Gpa1p does not directly require the presence of a classical Gβ-like subunit for its regulation.

Cpc2p is a WD-repeat protein and an ortholog of the *S. cerevisiae* protein Asc1p. *cpc2* encodes a 314 amino acids peptide, with a predicted molecular mass of 34.6 kDa and, analogous to Asc1p and Gnr1p, contains 7 predicted WD-repeats. Using EMBOSS Needle [30], we determined that Cpc2p-Asc1p share 67% similarity, while Cpc2p-Gnr1p only share 28%. Further, relatively high sequence alignment (64%) was also identified between Cpc2p and mammalian RACK1. For a more detailed structural analysis, we used *SWISS-MODEL Repository* [31], [32] to illustrate the multi-domain nature of the Cpc2p peptide, highlighting the 7 WD repeats (Figure 1B). Interestingly, many WD-repeat proteins contain a specific (up to 600 amino acids) C-terminal extension [33] although this motif does not seem to be present in Cpc2p.

Despite Cpc2p not appearing to be a negative regulator of pheromone signaling, it clearly performs a role in modulating the response. We therefore sought to determine what role Cpc2p may play upon both mitotically growing wild type (JY448; *cyr1^+^*, *sxa2* ^−^) and pheromone-responsive (JY546; h^−^, *cyr1* ^−^, *sxa2>lacZ*) cells. It should be noted that to enable quantitative characterization of the extent of pheromone-responsiveness we use strains defective for Cyr1p, which generates a genotype that mimics starvation and produces a more diminished cell size [34], [35] (Figure 1C).

For wild type mitotically growing Cyr1p expressing cells, disruption of the *cpc2* ORF resulted in cells displaying a slightly decreased size at the point of cell division (Wild type (JY448)  = 12.16±0.44 µm; *cpc2* ^−^ (JY1712)  = 11.32±0.21 µm) although this was not statistically significant (p>0.5; n  = 400). A more significant effect (p<0.1, n = 400) was observed upon overexpression of Cpc2p (*oe-cpc2p^+^*) with cells displaying a decreased size at the point of cell division (*oe-cpc2^+^* (JY1711)  = 10.8±0.11 µm). As mentioned above cells lacking Cyr1p display a smaller size at division (Figure 1D). Both the deletion or overexpression of the *cpc2* ORF from these strains increased the overall cell size (Wild type (JY546)  = 9.26±0.29 µm; *cpc2* ^−^ (JY1628)  = 10.6±0.64 µm; *oe-cpc2^+^* (JY1578)  = 10.38±0.10 µm) however these changes were not found to be statistically significant (*cpc2* ^−^ (JY1628) = p>0.5, n  = 400; *oe-cpc2^+^* (JY1578) = p>0.5, n  = 400). Some of these sizes are different to what maybe predicted from previous studies [18] but may arise due to the genetic backgrounds we have used compared to others. Indeed while all of the cell sizes at division in this study appear to be slightly smaller than accepted within the field (*S. pombe* cells generally undergo cell division at a size of between 13-14 µm), it should be noted that the growth media used in our assays is a variant of the classical EMM as described by Davey *et al.,* 1995 [36]. This media places a higher nutritional demand on the cells and as a result causes the cells to undergo division at a smaller size. Indeed it has been documented that nutritional reduction of growth rate results in decreased cell size [37].

We next sought to determine the effects upon the cell cycle of deleting, or overexpressing Cpc2p, using the previously described strains (Figure 1E). Deletion of Cpc2p slightly increased the percentage of cells within a population contain multiple-septa (regardless of the presence or absence of Cyr1) and is consistent with previous reports [26]. Interestingly, and somewhat counter-intuitive, this effect was more pronounced upon overexpression of Cpc2p regardless of the presence (Wild type (JY448) = <0.1%; *oe-cpc2^+^* (JY1711)  = 10±0.2% - p = <0.0001, n  = 4) or absence of Cyr1p (Wild type (JY546) = <0.1%; *oe-cpc2^+^* (JY1578)  = 10±0.15% - p = <0.0001, n  = 4). These results are consistent with Cpc2p inhibiting the activity of Ran1p/Pat1p resulting in an increase in Ste11p activity. Further, Cpc2p and Msa2p activity are tightly coordinated and thus increased Cpc2p expression may reduce the ability of Msa2p to stabilize *cdc4* mRNA, causing defects in cell division.

Deletion of *cpc2* renders cells of both mating types sterile (Figure 1F). It should be noted that all strains used in the quantitative mating assays express *sxa2* since strains lacking Sxa2p are sterile [28]. Significantly, increasing Cpc2p expression leads to a ∼1.5 fold increase in mating efficiency (observed for both mating types).

**Figure 2:**
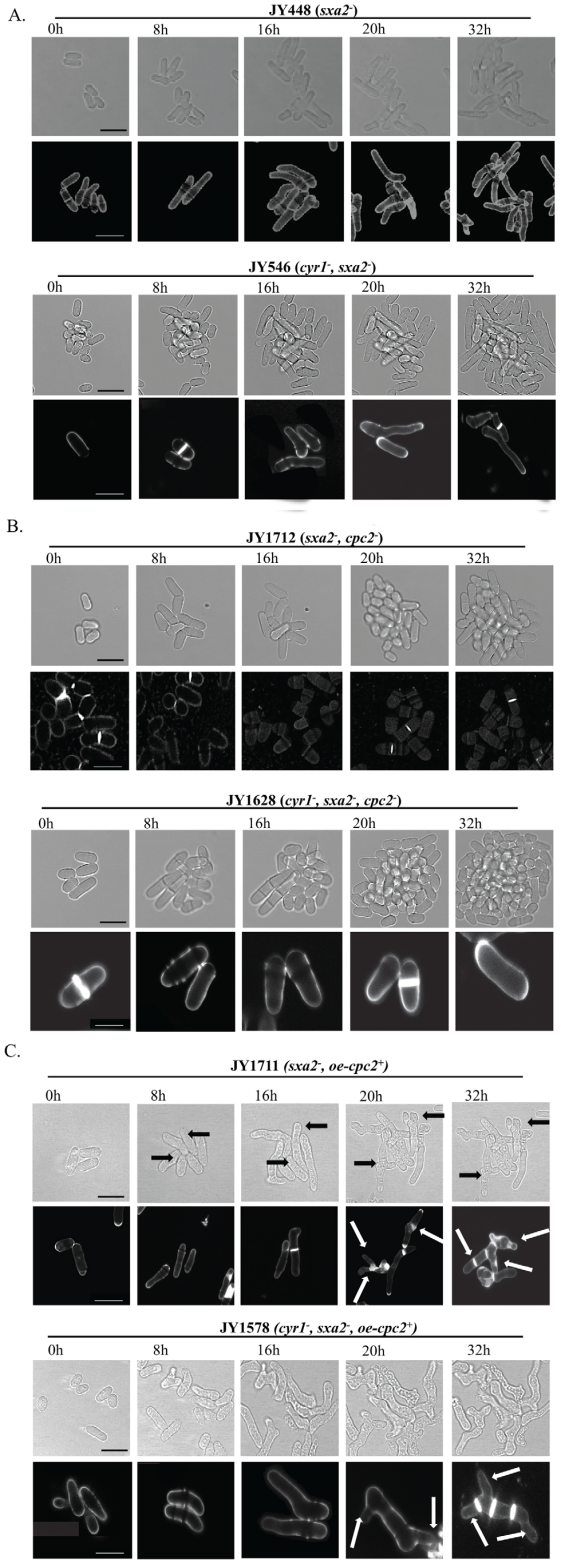
Cpc2p has profound morphological effects upon pheromone-stimulated cells. The strains (A) JY448 (h^−^, sxa2^−^) and JY546 (h^−^, cyr1^−^, sxa2>lacZ); (B) JY1712 (h^−^, sxa2^−^, cpc2^−^) and JY1628 (h^−^, cyr1^−^, sxa2>lacZ, cpc2^−^); (C) JY1711 (h^−^, sxa2^−^+oe-cpc2^+^), JY1578 (h^−^, cyr1^−^, sxa2>lacZ+oe-cpc2^+^), were grown to mid-exponential phase over 32 h in minimal media. Cells were then imaged using bright field microscopy on pads containing 10 µM of pheromone (see methods). Cells were also stained with calcofluor white (lower panels A-C) to visualize septation. Scale bars 10 µm. Prolonged exposure to pheromone for cells overexpressing Cpc2p (oe-cpc2^+^) results in multiple projection tips and a failure to undergo cytokinesis. Cells lacking Cpc2p fail to generate the classical shmoo formation as observed for control cells.

Having established a role for Cpc2p during the mating response, we next sought to provide a molecular explanation for these effects. During mating cells undergo a unidirectional elongation towards a potential partner (termed a shmoo) [38]. Our strains (either expressing or deleted for *cyr1*) lack Sxa2p and therefore display abnormally long conjugation tubes following prolonged exposure (>8 h) to high pheromone concentrations (10 µM) in the absence of a sufficient nutrient source (Figure 2A) [28]. After approximately 10-12 h these cells appear to resume mitotic division, before generating a second pheromone dependent-response. Strains deleted for *cpc2* failed to generate any morphological response to the presence of the pheromone and continued mitotic growth (Figure 2B). Strains overexpressing Cpc2p, when grown in the absence of nitrogen and treated with pheromone, initially generated conjugation tubes of equivalent size to wild type cells however, they failed to undergo division. Instead, after >16 h, cells displayed very specific cell branching activity (Figure 2C - arrows) but did not appear to lose viability. Moreover, following prolonged pheromone exposure (>30 h) many cells contained an increased number of septa, suggesting an inability to efficiently divide. As described previously, these data are consistent with Cpc2p having pleiotropic effects on the ability of Msa2p to regulate *cdc4* mRNA.

**Figure 3:**
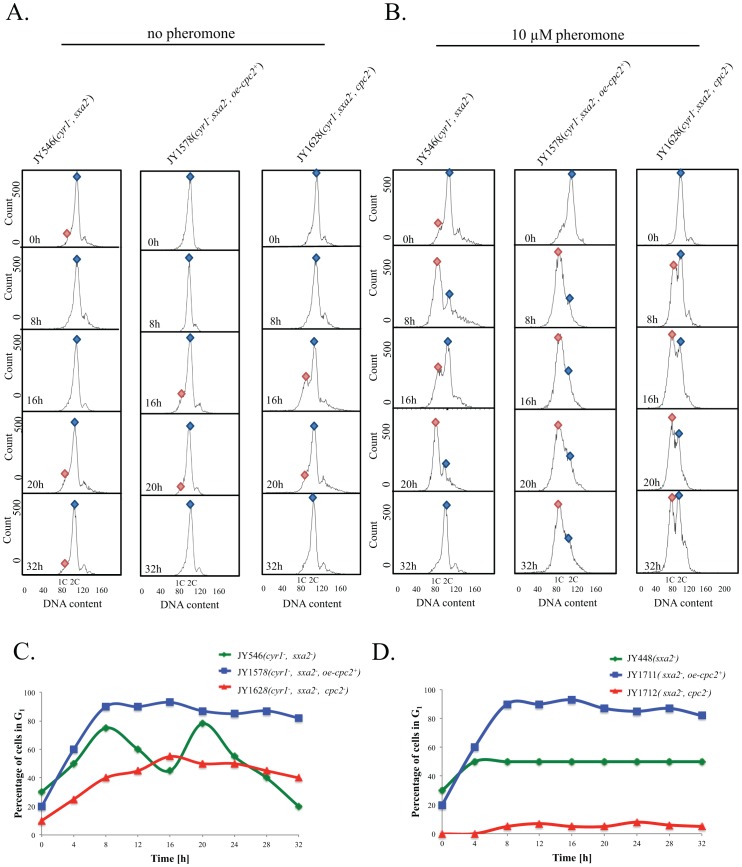
Cpc2p control the G1/S transition in pheromone-stimulated cells. (A) The strains JY546 (h^−^, cyr1^−^, sxa2>lacZ), JY1578 (h^−^, cyr1^−^, sxa2>lacZ, +oe-cpc2^+^) and JY1628 (h^−^, cyr1^−^, sxa2>lacZ, cpc2^−^) were grown in minimal medium and (B) minimal media containing 10 µM of pheromone for the times indicated. Cells were harvested and fixed prior to staining with propidium iodide prior to analysis using flow cytometry (see methods). The proportion of cells exhibiting 1C or 2C DNA content was determined using FACSDiva v4.1 software for the assigned gates indicated by the blue and red shapes. (C) The percentage of cells containing a 1C content (arrested in G_1_) as determined from B. Cells containing an additional content of Cpc2 fail to desensitize following pheromone stimulation and remain arrested for the time frame analyzed. Cells lacking Cpc2p fail to generate a significant arrest in G_1_ following exposure to 10 µM pheromone. (D) The percentage of cells from the strains JY448 (h^−^, sxa2^−^), JY1711 (h^−^, sxa2^−^+oe-cpc2^+^), JY1712 (h^−^, sxa2^−^, cpc2^−^) containing a 1C content (arrested in G_1_) following nitrogen starvation and stimulation with 10 µM pheromone.

The observation that cells overexpressing Cpc2p appear to elongate from multiple tips when exposed, for prolonged periods to pheromone, led us to hypothesize that these strains may exhibit a protracted pheromone-induced G_1_ cell cycle arrest. To address this, we used flow cytometry to analyze the response of cells when stimulated with pheromone. As mentioned previously, the strain JY546 lacks adenylate cyclase [34], and this results in an increased number of cells, within a population (∼20-25%) containing a single 1C content of DNA when analyzed using flow cytometry reflecting modification of the mitotic cell cycle in these strains (Figure 3C). Deletion of *cpc2* from strains lacking *cyr1* reduces the percentage of cells within a population containing a single 1C content of DNA, while overexpression did not have a significant effect (p>0.5, n = 3).

Following exposure to 10 µM pheromone, the proportion of wild type (JY546) cells with a 1C content of DNA steadily increased reaching a maximum (after 8 h) before decreasing over the subsequent 8 h time-frame. This cycle was repeated for the following 16 h time period. These two phases reflect the morphological images shown in Figure 2A and confirm that cells lacking Cyr1p only respond for a fixed period (∼10–12 h) before resuming mitotic division. In contrast, overexpression of Cpc2p results in an accelerated (compared to wild type) increase in the percentage of cells exhibiting a 1C content of DNA (90%, n = 3) following exposure to pheromone (Figure 3B and 3C). Consistent with the morphological data presented in Figure 2C, these cells failed to regain a 2C DNA content over the time frame analyzed. Interestingly, the strain lacking *cpc2* (JY1628) did show an increase (from 10% to 55%) in the percentage of cells containing a single 1C content of DNA following pheromone stimulation. This did not however coincide with an increase in cell size (Figure 2B) suggesting these cells can detect the presence of the pheromone, undergo an arrest but fail to initiate shmoo formation.

We next sought to observe if similar effects upon cell cycle regulation was observed for strains expressing Cyr1p, when nitrogen starved and exposed to pheromone (Figure 3D). Significantly, cells overexpressing Cpc2p appeared to increase the number of cells entering and remaining in a G_1_ arrest. Unlike the strains lacking *cyr1*, nitrogen starvation prevents cells from regaining their 2C complement and therefore they remain arrested in G_1_ (reviewed [13]). Overexpression of Cpc2p increased the percentage of cells within the population that contained a single 1C content following pheromone stimulation. Interestingly however, strains lacking *cpc2* did not appear to display any significant arrest in G_1_. This is in contrast to the data observed with *cyr1* ^−^ strains and highlights potential differences that this nutritional selection may enforce on *S. pombe* cells. Moreover the data would suggest that the regulatory mechanism utilized by Cpc2p and Cyr1p to control G_1_ arrest are different. It is entirely probable that the increased percentage of cells that initially contain a 1C content in a *cyr1* ^−^ background amplifies any potential arrests observed for strains following pheromone stimulation.

Overall, these data suggest that Cpc2p proactively induces the pheromone-dependent G_1_ arrest and following prolonged exposure, cells overexpressing Cpc2p are unable to become desensitized to pheromone and remain arrested in the G_1_ phase of the cell cycle although the precise mechanism for this protracted G_1_ arrest is unknown but maybe due, in part, to Cpc2p partially inhibiting Ran1p/Pat1p. Cells lacking *cpc2* do not appear to generate a typical pheromone-induced G_1_ arrest and this is consistent with the lack of shmoo formation observed previously (Figure 2B).

Mammalian RACK1 has been reported to, in mitotically growing cells mouse NIH3T3 cells, delay their entry into S phase [21], [39]. Further, it has been suggested that RACK1 suppresses *cpc2* defects in *S. pombe* cells [17]. We therefore sought to determine the effects on pheromone-induced responses in our strains expressing mammalian RACK1. Cells lacking an endogenous copy of the *cpc2* were transformed with an inducible plasmid expressing mammalian RACK1 and their dose-dependent response to pheromone determined using the *sxa2>lacZ* reporter. As has previously been suggested [17], mammalian RACK1 was able to functionally complement for the loss of Cpc2p, with cells generating a near normal pheromone-dependent response profile (Figure S3A). Further, upon investigation of the DNA content of cells expressing RACK1, when exposed to 10 µM pheromone, we observed a prolonged G_1_ arrest (Figure S3B) analogous to that described for increased Cpc2p expression (compare Figure 3C and 3D with Figure S3B). Taken together, these data highlight the functional similarities between Cpc2p and RACK1 in modulating the cells ability to exit pheromone-induced G_1_ phase of the cell cycle.

Having established that pheromone treated cells overexpressing Cpc2p display a protracted G_1_ arrest, we sought to determine if this was as a direct result of cells failing to enter S phase. *S. pombe* relies on a single cyclin-dependent kinase (CDK) Cdc2p, to regulate and control mitotic cell-cycle events. Cdc2p is controlled by association with B-type cyclins such that, Cdc2p-Cdc13p act as the mitotic kinase [40], [41], and Cdc2p-Cig2p the G_1_-S kinase [42]. In *S. pombe* cells, Rum1p, the sole cyclin-dependent kinase inhibitor (CKI), plays an important role in regulating both Cdc2p-Cig2p and Cdc2p-Cdc13p (over expression of Rum1p induces polyploidy due to the bypass of M-phase [43]). During anaphase, Rum1p starts to accumulate, becomes stable and consistent during G_1_, and diminishes in S-phase [43]. In addition, its expression is increased upon exposure to pheromone (http://www.pombase.org/). Via its regulating activity of Cdc2p-Cig2p, Rum1p functions as the main controlling agent for maintaining the G_1_ phase of the cell cycle [40]-[42], [44]. We therefore generated a pheromone-responsive reporter strain lacking Rum1p (JY1520; h^−^, *cyr1* ^−^, *sxa2>lacZ, rum1* ^−^) capable of overexpressing Cpc2p.

**Figure 4:**
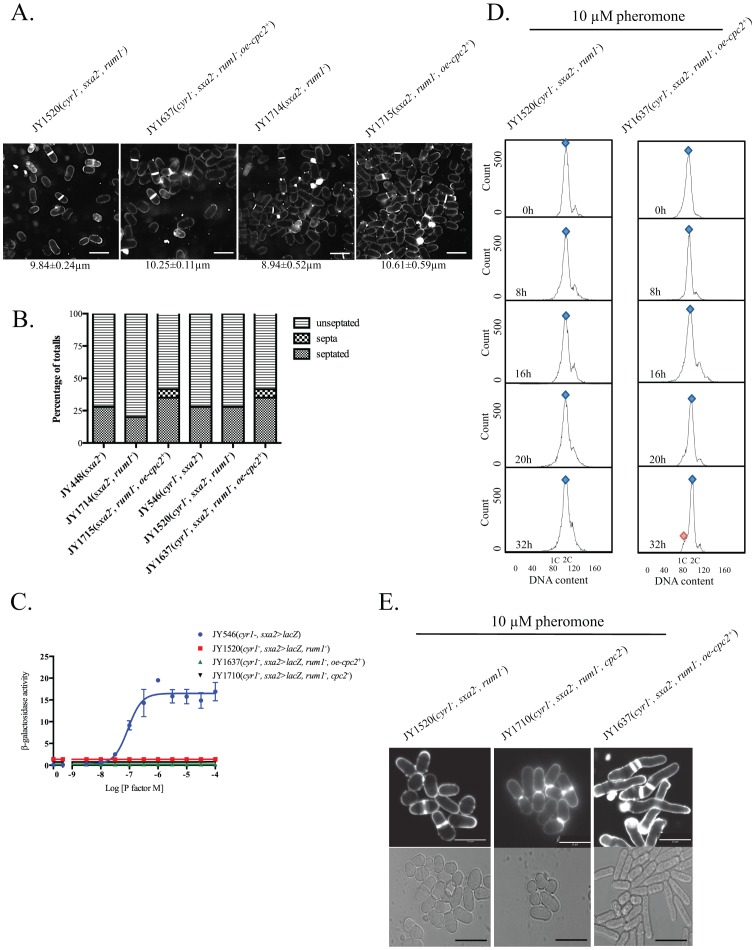
Overexpression of cpc2 mediates its pheromone effects in a G1-dependent manner. (A) Cell morphology and size, at division (micrometers ± S.D.) for strains JY1520 (h^−^, cyr1^−^, sxa2>lacZ, rum1^−^), JY1637 (h^−^, cyr1^−^, sxa2>lacZ, rum1^−^+ oe-cpc2^+^), JY1714 (h^−^, sxa2^−^, rum1^−^) and JY1715 (h^−^, sxa2^−^, rum1^−^+oe-cpc2^+^) grown in minimal medium at 29°C and stained with calcofluor. Scale bars 10 µm. (B) Number of non-septated, septated and multiple septa containing cells for the strains JY448 (h^−^, sxa2^−^), JY1714 (h^−^, sxa2^−^, rum1^−^), JY1715 (h^−^, sxa2^−^, rum1^−^+oe-cpc2^+^), JY546 (h^−^, cyr1^−^, sxa2>lacZ), JY1520 (h^−^, cyr1^−^, sxa2>lacZ, rum1^−^ ) and JY1637 (h^−^, cyr1^−^, sxa2>lacZ, rum1^−^+oe-cpc2^+^) were determined from 400 individual cells. Values shown correspond to the percentage of the total population. Cells were stained with calcofluor white, to enable visualization of septum material. (C) Pheromone-dependent transcription for the strains JY546, JY1520, JY1637 and JY1710 (h^−^, cyr1^−^, sxa2>lacZ, rum1^−^, cpc2^−^) was determined using the sxa2>lacZ reporter. Cells were stimulated with pheromone for 16 h in minimal media and assayed for β-galactosidase production using ONPG. Activity is expressed as OD_420_ units per 10^6^ cells. Values are means of triplicate determinations ± S.E.M. (D) The strains JY1520 and JY1637 were grown in minimal medium containing 10 µM of pheromone for the times indicated. Cells were harvested and fixed prior to staining with propidium iodide prior to analysis using flow cytometry (see methods). The proportion of cells exhibiting 1C or 2C DNA content was determined using FACSDiva v4.1 software for the assigned gates indicated by the blue and red shapes (E) The strains JY1520, JY1710 and JY1637 were grown to mid-exponential phase over 32 h in minimal media. Cells were then stained with calcofluor white to visualize septation (top panel) and imaged bright field microscopy (bottom panel) after 32 h exposure to pheromone. Scale bars 10 µm.

Cells lacking Rum1p displayed a smaller size (irrespective of the expression of Cyr1p) than wild type strains (Figure 4A), although their morphology appeared normal. Further, there were no observable defects in the levels of septation (Figure 4B) and/or growth rates for the strain lacking Rum1p. Overexpression of Cpc2p in strains lacking Rum1p resulted in a slight increase in cell size (Figure 4A) and a 7-fold increase in the occurrence of multiple septa (Figure 4B).

We next investigated the pheromone-dependent responses of cells lacking Rum1p with and without additional Cpc2p (Figure 4C). Cells lacking Rum1p failed to generate a dose-dependent response to stimulation with pheromone and this was not recovered by the overexpression of Cpc2p (JY1637). Flow cytometry analysis revealed that both these populations (JY1520 (*rum1^−^*) and JY1637 (*rum1^−^*+*oe-cpc2^+^*)) contained a 2C content of DNA regardless of the presence of 10 µM pheromone (Figure 4D), suggesting that cells failing to reside in the G_1_ phase of the cell cycle for an extended period, are not subjected to pheromone-induced Cpc2p regulation of the G_1_/S transition.

Significantly, bright field microscopy images of our Rum1p deleted strains overexpressing Cpc2p, following exposure to 10 µM pheromone (for 32 h) indicate the presence of elongated cells compared to the non-stimulated populations (Figure 4E). These effects were not observed in a strain lacking both Rum1p and Cpc2p (JY1710; h^−^, *cyr1^−^*, *sxa2>lacZ, cpc2^−^, rum1^−^*). The pheromone-induced elongate cells in the *rum1* deleted strain overexpressing Cpc2p showed considerable similarity to the classical shmoo morphology described in Figure 2A. The presence of pheromone-induced elongated cells in a strain lacking Rum1p strain suggests the possibility that *S. pombe* cells can generate a G_1_-independent morphological response to pheromone. This data would suggest that pheromone induces shmoo formation at the same time as a G_1_ arrest. To the best of our knowledge, these observations represent the first demonstration of a pheromone-induced cell elongation for an *S. pombe* population that fails to enter G_1_.

Following our observations that Cpc2p may play a role in modulate cells passage into S phase, we sought to provide a mechanistic interpretation to our data. The failure of cells lacking Rum1p to enter a protracted G_1_ arrest suggests that Cpc2p performs its role by over-activation of the Ste11p-regulated pheromone-response pathway. Following pheromone activation of cell surface receptors, Gpa1p propagates the response by activating Ras1p. This leads to stimulation of a MAP kinase cascade and activation of Ste11p. Further, Spk1p also phosphorylates an unknown negative regulator of Msa2p [25], reducing it’s activity leading to an increase in Ste11p translation therefore reinforcing the response.

**Figure 5:**
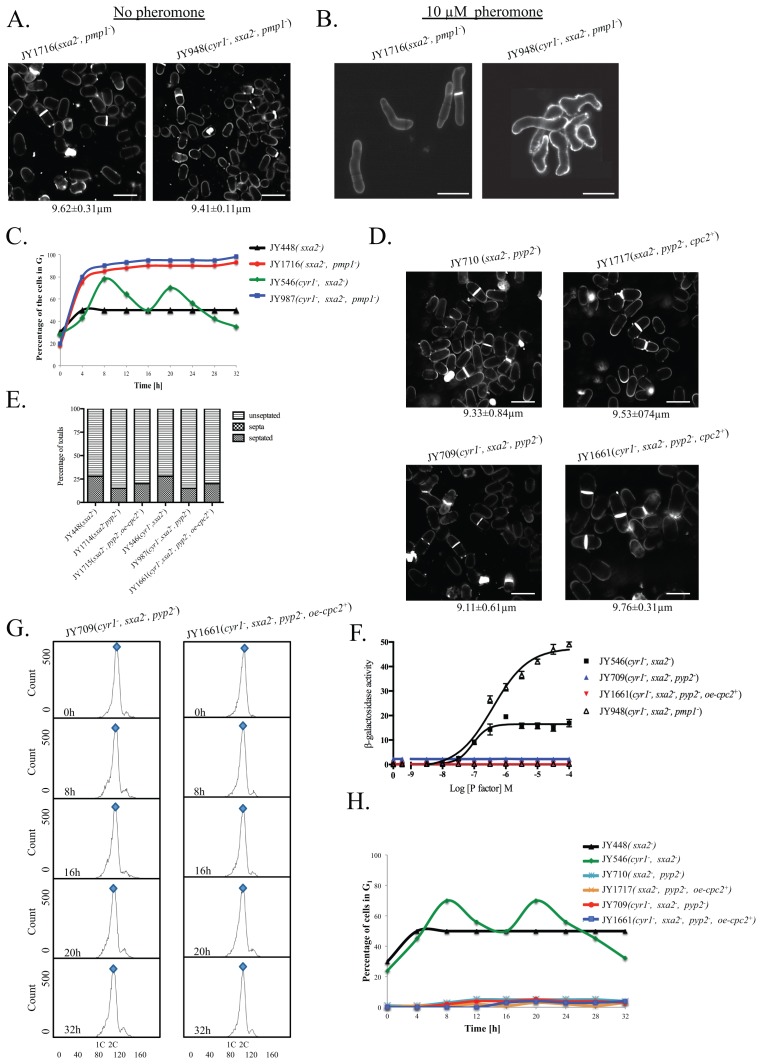
Overexpression of Cpc2 in pheromone stimulated cells mimics prolonged pheromone stimulation. (A) Cell morphology and size, at division (micrometers ± S.D.) for the strains JY1716 (h^−^, sxa2^−^, pmp1^−^) and JY948 (h^−^, cyr1^−^, sxa2>lacZ, pmp1^−^) grown in minimal medium at 29°C and stained with calcofluor white. (B) Strains from A strains were exposure to 10 µM of pheromone for 32 h and stained with calcofluor white. (C) The percentage of cells containing a 1C content (arrested in G_1_) for the strains JY448, JY1716, JY546 and JY948 as determined using flow cytometry. Cells lacking Pmp1p show a failure to exit from a G_1_ arrest analogous to strains where the cpc2 ORF has been deleted. (D) Cell morphology and size, at division (micrometers ± S.D.) for strains JY710 (h^−^, sxa2^−^ pyp2^−^) and JY1717 (h^−^, sxa2^−^, pyp2^−^+oe-cpc2^+^) grown in minimal medium at 29°C and stained with calcofluor white (top panel). Cell morphology and size at division (micrometers ± S.D.) for the strains JY709 (h^−^, cyr1^−^, sxa2>lacZ, pyp2^−^) and JY1661 (h^−^, cyr1^−^, sxa2>lacZ, pyp2^−^+oe-cpc2^+^) grown in minimal medium at 29°C and stained with calcofluor white (bottom panel). (E) Numbers of non-septated, septated and multiple septa containing cells for the strains JY448, JY1714, JY1715, JY546, JY987 and JY1661 were determined from 400 individual cells. Values shown correspond to the percentages of the total population. Cells were stained with calcofluor white, to enable visualization of septum material. (F) Pheromone-dependent transcription for the strains JY546, JY709, JY1661 and JY948 was determined using the sxa2>lacZ reporter. Cells were stimulated with pheromone for 16 h in minimal media and assayed for β-galactosidase production using ONPG. Activity is expressed as OD_420_ units per 10^6^ cells. Values are means of triplicate determinations ± S.E.M. (G) The strains JY709 and JY1661 were grown in minimal medium containing 10 µM of pheromone for the times indicated. Cells were harvested and fixed prior to staining with propidium iodide prior to analysis using flow cytometry (see methods). The proportion of cells exhibiting 1C or 2C DNA content was determined using FACSDiva v4.1 software for the assigned gates indicated by the blue and red shapes. (H) The percentage of cells containing a 1C content (arrested in G_1_) as determined for the strains JY448, JY546, JY710, JY1717, JY709 and JY1661.

The promiscuous dual-specificity phosphatase Pmp1p, has among other roles, been suggested to negatively modulate the action of Spk1p [45]. Deletion of Pmp1p results in Spk1p remaining activate for longer so prolonging Ste11p activation [45]. Thus, we sought to determine if similar cellular defects were observed in Pmp1p deleted cells to that observed for cells overexpressing Cpc2p. Morphological analysis of a strain lacking Pmp1p, (JY948; h^−^, *cyr1^−^*, *sxa2>lacZ, pmp1^−^* and JY1716; h^−^, *sxa2^−^, pmp1^−^*) when stimulated with 10 µM pheromone for 32 h, reveals cells that displayed extended conjugation tubes and multiple projection tips (Figure 5A and 5B). Further, Pmp1p depleted cells exhibit an elevated maximal response to pheromone (Figure 5F), and flow cytometry analysis suggests that these cells fail to exit from a pheromone-induced G_1_ arrest (Figure 5C). These results are consistent with that observed for overexpression of Cpc2p, and would suggest that Cpc2p could be acting to increase MAP kinase/Ste11p activity in response to pheromone.

It is of interested to note that Cpc2p does not perform a direct role in modulating the expression levels of Pmp1p but in contrast, it has been reported that Cpc2p regulates the activity of two other MAP kinase phosphatases Pyp1p and Pyp2p [18]. To date, little information has been published relating to potential roles of Pyp1p and Pyp2p in relation to pheromone signaling. Based upon our results with deletion of *pmp1* we sought to evaluate the role of Cpc2p modulation of these two phosphatases with respect to pheromone transduction. Unfortunately, despite numerous attempts the concurrent deletion of *pyp1* and *cyr1* appears synthetically lethal (Didmon and Davey unpublished). As a result we have only been able to investigate the role of Pyp2p.

Cells lacking Pyp2p (JY709; h^−^, *cyr1^−^*, *sxa2>lacZ, pyp2^−^*) and (JY710; h^−^, *sxa2,^−^ pyp2^−^*) have a slightly reduced size (Figure 5D) that is not significantly increase upon overexpression of Cpc2p. Interestingly, and in contrast to all other strains tested within this study, overexpression of Cpc2p did not induce an increase in septa formation (Figure 5E). It has been reported that cells overexpressing Pyp2p exhibit a delay in mitosis [46], and which appears analogous to the overexpression of Cpc2p [18]. Since Cpc2p has been documented to modulate the expression of Pyp2p, the removal of *pyp2* renders Cpc2p activity redundant. Finally, irrespective of the presence of excess Cpc2p, cells lacking Pyp2p fail to respond to pheromone (Figure 5F) and appear to contain a 2C DNA content at all times (Figure 5G and 5H). These results are similar to the Rum1p disruption, however cells lacking Pyp2p fail to generate a G_1_-independent morphological response to the presence of the pheromone.

## Discussion

The eukaryotic scaffold protein RACK1 (a WD-repeat protein) is involved in a wide range of signal transduction pathways including cell cycle progression. The fission yeast ortholog Cpc2p has been reported to modulate the stress-response pathway and positively regulates the G_2_/M transition at the ribosomal level [26]. Here we have described the role of Cpc2p in modulating the pheromone-response pathway. In a previously described yeast 2-hybid screen for interacting partners of Gpa1p, we identified Cpc2p [16]. Based upon the similarity of Cpc2p to the Asc1p (a Gβγ-like subunit for Gpa2p involved in the glucose-sensing pathway from budding yeast) [10] we investigate the potential for Cpc2p to act as the Gβγ-subunit within the pheromone-response pathway in fission yeast. Despite performing a significant role in pheromone-mediated signaling, Cpc2p does not appear to regulate Gpa1p and attempts to validate this interaction *in vivo* proved unsuccessful. Indeed, it is highly probable that Gpa1p does not require a chaperone to target it to the plasma membrane. Gpa1p contains a consensus MG_2_XXXS_6_ myristoylation sequence [47], and a downstream cysteine, which is likely to be a target for palmitoylation, so facilitating association with membranes. Moreover, mutational analysis suggests that the N-terminal first 40 amino acids of Gpa1p are necessary and sufficient to enable its association with membrane structures (Figure S2).

Our data has confirmed that Cpc2p performs a role in regulating sexual differentiation in *S. pombe*. Cells lacking Cpc2p display little change in the transcription levels of *ste11* mRNA despite but are sterile [25]. Interestingly, *cpc2* depleted cells do however, display decreased reduce protein concentration of Ste11p that is insufficient to induce downstream targets. Our studies have utilized a pheromone-reporter strain (*sxa2>lacZ*) under the direct control of Ste11p (Figure 1A) so validating the notion that *cpc2^−^* cells have reduced Ste11p activity.

Although Cpc2p does not appear to function as a Gβ-like subunit in *S. pombe*, our results suggest that in a pheromone-stimulated environment, overexpression of Cpc2p causes significant cellular effects including; defects in cell integrity, increased mating efficiency, increased septum formation, reduced cell cycle progression and increased cell morphological defects. Significantly, Cpc2p appears to actively promote and prolong the pheromone-induced G_1_ arrested state, preventing cells from adapting to the presence of the pheromone and resuming mitotic growth. Moreover, cells expressing RACK1 display the same cell cycle progression defect when subjected to pheromone stimulation, confirming functional similarity between these two orthologs.

The precise mechanism by which Cpc2p protracts the G_1_ arrest upon pheromone stimulation remains to be determined. Initially, it was tempting to speculate that this effect was due to the Cpc2p interaction with Msa2p. Indeed, upon pheromone stimulation Spk1p phosphorylates a negative regulator of Msa2p allowing production of Ste11p. However, Msa2p is expected to negatively regulate Cpc2p since it has been demonstrated that the *cpc2* null strain is phenotypically epistatic to an *msa2* deleted strain [22]. It is more plausible that the effects we observe following overexpression of Cpc2p are due to its role as an inhibitor of the essential kinase, Ran1p/Pat1p which has been reported to act by phosphorylating Mei2p so regulating sexual differentiation [48], [49]. Cpc2p has been demonstrated to bind to Ran1p/Pat1p resulting in a change in its cellular localization causing a loss of activity [17]. The partial inhibition of Ran1p/Pat1p in starved haploid cells leads to a G_1_ cell cycle arrest. This is consistent with our observations for cells lacking Cpc2p, which are sterile and fail to exhibit a pheromone-induced G_1_ arrest. Moreover, it has been demonstrated that Ran1p/Pat1p phosphorylates Ste11p to down regulate its activity [50]. Thus overexpression of Cpc2p will lead to enhanced inhibition of Ran1p/Pat1p so preventing negative regulation of Ste11p [51] and prolonging the G_1_ arrest. Further experimental work will be required to fully dissect the precise role that Cpc2p performs in modulating Ran1p/Pat1p activity and consequently Ste11p.

The pheromone-induced effects of Cpc2p are potentially re-enforced through the action of Msa2p. It has been suggested that upon pheromone stimulation, Spk1p phosphorylates an, as yet, unidentified negative regulator of Msa2p, so allowing increased production of Ste11p [25]. Overexpression of Cpc2p results in enhanced activation of Ste11p and coincides with abnormal morphologies and defects in cytokinesis (probably relating from reduced Cdc4p activity). Both these effects are consistent with a reduction in Msa2p activity since it negatively regulates Ste11p translation and also stabilizes *cdc4* mRNA [22]. Cpc2p modulates the activity of Atf1p and acts as a positive translational regulator of mRNA for Pyp1p and Pyp2p activity so reducing Pmk1p activity. This consequently decreases Msa2p activity.

Further to its roles in regulating sexual development through modulating Ran1p/Pat1p activity, Cpc2p has been described as being associated with the 40S ribosomal subunit so modulating gene transcription [27]. Paul and colleagues provided evidence to suggest that Cpc2p, through association with Moc2p and Rpl32-2p, may act to increase sexual differentiation [52]. Specifically, Rpl32-2p expression was shown to increase upon nitrogen starvation, analogous to Ste11p levels. In addition, deletion of *moc1* or *moc3* results in reduced *ste11* expression. Indeed it has been suggested that all Moc proteins and Cpc2p might act as a translational regulator involved in controlling sexual differentiation. Thus it is apparent that Cpc2p may play numerous roles in regulating the pheromone response by modulating *ste11* gene expression.

Previous investigations of Cpc2p activity within *S. pombe* have suggested that, in contrast to a role in modulating the G_1_/S transition, that Cpc2p acts to positively regulate the G_2_/M transition for mitotically growing cells [26]. However, as the authors themselves highlight, *S. pombe* cells spend most of their time (about three-quarters of the cell cycle) in G_2_ whereas for mammalian cells the longest phase of the cell cycle is G_1_ [26]. However, the addition of pheromone to *S. pombe* cells (subjected to nutrient limitation or removal of *cyr1*) causes them to initiate the meiotic mating-response pathway, resulting in cells remaining in the G_1_ phase of the cell cycle for longer. Under these conditions addition of exogenous RACK1/Cpc2p results in a protraction of the G_1_ arrest and prevents a return to mitotic growth. By the addition of pheromone to nitrogen starved *S. pombe* cells these now mimic mammalian cells by exhibiting an extended G_1_ so enabling RACK1/Cpc2p to suppress G_1_/S transition. These observations were confirmed by removal of Rum1p (the single CKI in fission yeast) or Pyp2p (MAPK phosphatases) both of which results in cells failing to enter G_1_ and consequently, were unaffected by the addition of exogenous Cpc2p. By manipulating the duration of the G_1_ phase of the cell cycle we have uncovered a subtle role that Cpc2p plays in *S. pombe.*

## Materials and Methods

### Strains, Reagents and General Methods

Fission yeast strains used in this study are listed in Table 1. Culture media used was YE - yeast extract for routine cell growth, amino acid (AA) medium for auxotrophic selection and DMM – a defined minimal medium for selective growth and all the assays. DMM is a variant of EMM and contains little nutritional supplement for the yeast. As a consequence cells can appear slightly more rounded than wild type strains. Nutritional limitation has previously been described as reducing the rate of cell growth and size [37]. As described previously lithium acetate was used for transformation of yeast [29], [36]. Cell concentrations were determined using a Coulter Channelyser (Beckman Coulter, Luton, UK). DNA manipulations were performed by standard methods. Oligonucleotides were synthesized by Invitrogen Ltd. (Paisley, Scotland, UK). Amplification by the polymerase chain reaction (PCR) used *Pwo* DNA polymerase (from *Pyrococcus woesei*) according to the supplier’s instructions (Boehringer–Mannheim Biochemicals, Lewes, East Sussex, UK). All constructs generated by PCR were confirmed by sequencing.

**Table 1:** *S. pombe* strains used in this study.

Strain	Genotype	Source/reference
JY383	*mat1-P, Δmat2/3::LEU2^−^, leu1-32, ade6-M216, ura4-D18*	[56]
JY402	*mat1-M, Δmat2/3::LEU2^−^, ade6-M216, leu1-32 ura4-D18*	[57]
JY448	*mat1-M, Δmat2/3::LEU2^−^, leu1-32^−^, ade6-M216, ura4-D18, sxa2-D15*	[57]
JY546	*mat1-M, Δmat2/3::LEU2^−^, leu1-32, ade6-M216, ura4-D18, cyr1-D51, sxa2>lacZ*	[45]
JY709	*mat1-M, Δmat2/3::LEU2^−^, leu1-32, ade6-M216, ura4-D18, cyr1-D51, sxa2>lacZ, pyp2::ura4^−^*	This study
JY710	*mat1-M, Δmat2/3^−^::LEU2^−^, leu1-32, ade6-M216, ura4-D18, sxa2-D15, pyp2::ura4^−^*	This study
JY948	*mat1-M, Δmat2/3::LEU2^–^, leu1-32, ade6-M216, ura4-D18, cyr1-D51, sxa2>lacZ, pmp1::ura4^−^*	This study
JY1034	*mat1-M, Δmat2/3::LEU2^−^, leu1-32, ade6-M216, ura4-D18, cyr1-D51, sxa2>lacZ,* pREP3x-^1−40^Gpa1-GFP	This study
JY1314	*mat1-M, Δmat2/3::LEU2^−^, leu1-32, ade6-M216, ura4-D18, cyr1-D51, sxa2>lacZ, gnr1::ura4^−^*	[12]
JY1315	*mat1-M, Δmat2/3::LEU2^−^, leu1-32, ade6-M216, ura4-D18, cyr1-D51, sxa2>lacZ, gnr1::ura4^−^*, pREP3x-^1−40^Gpa1-GFP	This study
JY1316	*mat1-M, Δmat2/3::LEU2^−^, leu1-32, ade6-M216, ura4-D18, cyr1-D51, sxa2>lacZ,* pREP3x-Gnr1	This study
JY1317	*mat1-M, Δmat2/3::LEU2^−^, leu1-32, ade6-M216, ura4-D18, cyr1-D51, sxa2>lacZ, gnr1::ura4^−^*, pREP3x-Gnr1	This study
JY1520	*mat1-M, Δmat2/3::LEU2^−^, leu1-32, ade6-M216, ura4-D18, cyr1-D51, sxa2>lacZ, rum1::ura4* ^−^	This study
JY1578	*mat1-M, Δmat2/3::LEU2^−^, leu1-32, ade6-M216, ura4-D18, cyr1-D51, sxa2>lacZ,* pREP3x-Cpc2	This study
JY1628	*mat1-M, Δmat2/3::LEU2^−^, leu1-32, ade6-M216, ura4-D18, cyr1-D51, sxa2>lacZ, cpc2::ura4^−^*	This study
JY1629	*mat1-M, Δmat2/3::LEU2^−^, leu1-32, ade6-M216, ura4-D18, cyr1-D51, sxa2>lacZ, cpc2::ura4^−^*, pREP3x-^1−40^Gpa1-GFP	This study
JY1633	*mat1-P, Δmat2/3::LEU2^−^, leu1-32, ade6-M216, ura4-D18,* pREP3x-Cpc2	This study
JY1634	*mat1-M,Δmat2/3::LEU2^−^, leu1-32, ade6-M216, ura4-D18,* pREP3x-Cpc2	This study
JY1635	*mat1-P, Δmat2/3::LEU2^−^, leu1-32, ade6-M216, ura4-D18, cpc2::ura4* ^−^	This study
JY1636	*mat1-M,Δmat2/3::LEU2^−^, leu1-32, ade6-M216, ura4-D18, cpc2::ura4* ^−^	This study
JY1637	*mat1-M, Δmat2/3::LEU2^−^, leu1-32, ade6-M216, ura4-D18, cyr1-D51, sxa2>lacZ, rum1::ura4* ^−^, pREP3x-Cpc2	This study
JY1661	*mat1-M, Δmat2/3::LEU2^–^, leu1-32, ade6-M216, ura4-D18, cyr1-D51, sxa2>lacZ, pyp2::ura4^−^,* pREP3x-Cpc2	This study
JY1662	*mat1-M, Δmat2/3::LEU2^−^, leu1-32, ade6-M216, ura4-D18, cyr1-D51, sxa2>lacZ, cpc2::ura4* ^−^, pREP3x-RACK1	This study
JY1663	*mat1-M, Δmat2/3::LEU2^−^, leu1-32, ade6-M216, ura4-D18, cyr1-D51, sxa2>lacZ,* pREP3x-RACK1	This study
JY1710	*mat1-M, Δmat2/3::LEU2^−^, leu1-32, ade6-M216, ura4-D18, cyr1-D51, sxa2>lacZ, rum1::ura4^−^, cpc2::ura4^−^*	This study
JY1711	*mat1-M, Δmat2/3::LEU2^−^, leu1-32, ade6-M216, ura4-D18, sxa2-D15,* pREP3x-Cpc2	This study
JY1712	*mat1-M, Δmat2/3::LEU2* ^−^, *leu1-32, ade6-M216, ura4-D18, sxa2-D15, cpc2::ura4^−^*	This study
JY1713	*mat1-M, Δmat2/3::LEU2^−^, leu1-32, ade6-M216, ura4-D18, cyr1-D51, sxa2>lacZ, cpc2::ura4^−^,* pREP3x-Cpc2	This study
JY1714	*mat1-M, Δmat2/3::LEU2^−^, leu1-32, ade6-M216, ura4-D18, sxa2-D15, rum1::ura4^−^*	This study
JY1715	*mat1-M, Δmat2/3::LEU2^−^, leu1-32, ade6-M216, ura4-D18, sxa2-D15, rum1::ura4^−^,* pREP3x-Cpc2	This study
JY1716	*mat1-M, Δmat2/3::LEU2^−^, leu1-32, ade6-M216, ura4-D18, sxa2-D15, pmp1::ura4^−^*	This study
JY1717	*mat1-M, Δmat2/3::LEU2^−^, leu1-32, ade6-M216, ura4-D18, sxa2-D15, pyp2::ura4* ^−^, pREP3x-Cpc2	This study

### Disruption of Endogenous S. pombe cpc2

The upstream region of *cpc2* locus was amplified from *S. pombe* genomic DNA using sense oligonucleotide JO2836 (aaaTCTAGACTAGAGCATTATTCAAGATAAATTTC; position 954 to 928 relative to *cpc2* ATG, *Xba*I site underlined) and antisense oligonucleotide JO2837 (aaaGGATCCCCCCTTCACTGGTCGGGATGTC; position 35 to 3 relative to *cpc2* ATG, *Bam*HI site underlined). The downstream region of *cpc2* locus was amplified from *S. pombe* genomic DNA using sense oligonucleotide JO2838 (aaaGGATCCGGGAAA*TAA*GATTTTAATTGTTGTCCC; position 4 to 40 relative from *cpc2* STOP anticodon, *Bam*HI site underlined) and antisense oligonucleotide JO2839 (aaaCCCGGGGAACAACAACT*ATT*CAGCCACCCCCAGCGAAGG; position 444 to 485 relative from *cpc2* STOP anticodon (italics), *Sma*I site underlined). A 1.8 kb fragment of the *S. pombe ura4^+^* cassette was amplified using sense oligonucleotide JO1049 (CTGGATCCACCATGTAGCTACAAATCC) and antisense oligonucleotide JO1050 (CTGGATCCACCATGTAGTGATATTGAC). Both up and downstream PCR products were cloned respectively into pKS^+^ Bluescript (Stratagene) digested with *Bam*HI. The *ura4^+^* cassette (JD3725) was cloned into the unique *Bam*HI site within the *cpc2* ORF to create *cpc2*::*ura4^+^*. The strains JY448 (h^−^, *sxa2^−^*) and JY546 (h^−^, *cyr1^−^, sxa2>lacZ*) were transformed with the *Xba*I/*Sma*I fragment from JD3725 and integration of the *ura4* cassette selected by growth on medium lacking uracil. To enable consistent comparison with the parent strains, which were *ura4^−^*, the Ura4 ORF was subsequently deleted from the *cpc2* locus using a *Bam*HI digest of JD437 (pKS^+^ Bluescript containing the 5′ and 3′ un-translated regions of the *ura4* cassette separated by an *Eco*RV site). We have used similar techniques for disruption of other members of the *S. pombe* pheromone-response pathway [11]. Transformants were selected by growth on minimal media supplemented with 5-fluoro-orotic acid (FOA). The resultant strains produced were JY1712 (h^−^, *sxa2^−^, cpc2^−^*) and JY1628 (h^−^, *cyr1^−^*, *sxa2>lacZ, cpc2^−^*).

### Disruption of Endogenous Rum1

A similar two-step technique as described for *cpc2* was used for the disruption of *rum1*. Briefly, the *rum1* locus was amplified from *S. pombe* genomic DNA using sense oligonucleotide JO2658 (gggACTAGTTTTTAAATTCTAACATTAG; position -569 to -545 relative to *rum1* ATG, *Spe*I site underlined) and antisense oligonucleotide JO2659 (cccACTAGTTATTGAGAATAACAGAC; position 550 to 568 relative from *rum1* STOP anticodon, *Spe*I site underlined). This product was cloned into pKS^+^ Bluescript digested with *Pvu*II. The ORF was removed from this construct and replaced with a unique *Bgl*II site by inverse PCR to generate JD3674 using the antisense oligonucleotide JO2661 (AGATCT AGCGAACTGACAATCC; position -1 to -16 relative to *rum1* ATG, *Bgl*II site underlined) and sense oligonucleotide JO2660 (AGATCT CGCATTTTGTAATTGTGTTTG; position 10 to 31 relative from *rum1* STOP anticodon, *Bgl*II site underlined). The 1.8 kb *ura4^+^* cassette was cloned into the unique *Bgl*II site of JD3674 to create the *rum1*::*ura4^+^* contained within pKS^+^ Bluescript (JD3675). The strains JY1628 (h^−^, *cyr1^−^*, *sxa2>lacZ, cpc2^−^*) and JY546 (h^−^, *cyr1^−^, sxa2>lacZ*) were transformed with the *Spe*I fragment from JD3675 and integration of the *ura4* cassette selected by growth on medium lacking uracil. Removal of the Ura4 ORF from the locus of *rum1* was achieved as described for *cpc2*. The resultant strains produced were JY1710 (h^−^, *sxa2^−^, cpc2^−^, rum1^−^*), JY1520 (h^−^, *cyr1^−^*, *sxa2>lacZ, rum1^−^*).

### Disruption of Endogenous pmp1 and pyp2

We have previously described the generation of the strain JY723 that lacks *pmp1* (h^−^, *cyr1^−^*, *sxa2>lacZ, pmp1::ura4^+^*) [45]. Removal of the Ura4 ORF from this strain generated JY948 (h^−^, *cyr1^−^*, *sxa2>lacZ, pmp1::ura4^−^*). A similar two-step method was used to disrupt *pmp1* from JY448 (h^−^, *sxa2^−^*) generating JY1716 (h^−^, *sxa2^−^, pmp1::ura4^−^*). Disruption of *pyp2* was again accomplished using a two-step integration strategy. The *pyp2* locus was amplified using sense oligonucleotide JO842 (gggcagCTGTTCAACATCAATAGGCAA; position -528 to -508 relative to *pyp2* ATG, *Pvu*II site underlined) and antisense oligonucleotide JO843 (gggcagCTGGTAACAATGCAAATCAAC; position 531 to 551 relative from *pyp2* STOP anticodon) and cloned into pKS^+^ Bluescript digested with *Pvu*II. The Pyp2 ORF was removed from this construct and replaced with a unique *BamH*I site by inverse PCR to generate JD970 using the antisense oligonucleotide JO844 (ggggatccTTGAAAACACCTTGGAAGATG; position -16 to -36, relative to *pyp2* ATG, *Bam*HI site underlined) and sense oligonucleotide JO845 (ggggatccGATGACTTAACGAAACGACTG; position -7 to 14 relative from *pyp2* STOP anticodon). The 1.8 kb *ura4^+^* cassette was cloned into the unique *Bam*HI site of JD970 to create the *pyp2*::*ura4^+^* contained within pKS^+^ Bluescript (JD978). The strains JY448 (h^−^, *sxa2^−^*) and JY546 (h^−^, *cyr1^−^, sxa2>lacZ*) were transformed with the *Pvu*II fragment from JD978 and integration of the *ura4* cassette selected by growth on medium lacking uracil. Removal of the Ura4 ORF from the locus of *pyp2* was achieved as described for *cpc2*. The resultant strains produced were JY709 (h^−^, *cyr1^−^, sxa2>lacZ*, *pyp2^−^*) and JY710 (h^−^, *sxa2^−^, pyp2^−^*). All gene replacements were confirmed by PCR from genomic DNA.

### The Mating-response Assay

Cells were cultured in liquid DMM to a density of 5x10^6^ cells/ml. 200 µl of each mating type strain were mixed and harvested by centrifugation (2000 rpm for 3 min). Cells were suspended in 10 µl of sterile water and spotted onto low nitrogen containing DMM plates. Non-mixed controls were also spotted onto the plates. Following 72 h incubation at 29°C, each colony was collected from the spots and suspended in 1 ml of sterile water. Two separate 1 in 100 dilutions were then made from each 1 ml culture. One of these was plated onto separate YE plates at final dilution factors of 1 in 1,000 and 1 in 10,000 respectively. The other was placed in a 55°C heat block for 10 min to heat-inactivate everything except spores formed from mating events. This heat-treated sample was then also plated onto separate YE plates at final dilution factors of 1 in 1,000 and 1 in 10,000. Following 72 h incubation, the number of colonies on each plate was counted using a G-Box iChemi gel documentation system with GeneTool analysis software (Syngene, Cambridge, UK). The number of colonies as a percentage of the total (colony survival) can then be calculated for mated strains and controls, then the mating efficiency calculated as colony survival (mated strains) - colony survival (non-mated control strain).

### pREP Expression Constructs

pREP series of *S. pombe* vectors allows expression of genes under the control of the thiamine-repressible *nmt1* promoter [53]. pREP3x-Cpc2 has been kindly offered by Jose Cansado (University of Murcia, Spain). The ORF of mammalian RACK1 was amplified from a plasmid donated by Dorit Ron (University of California, USA) using the sense primer JO2996 (GCCACCATGGATTACAAGGAT), and the antisense primer JO1606 (*TTA* GCGGGTACCAATAGTCACCT) which contains the stop anticodon (italics). The PCR product was cloned into the unique *Eco*RV site of a modified pREP3x vector to generate (pREP3x-RACK1).

^1−40^Gpa1 was first amplified using a sense oligonucleotide beginning at the initiating ATG (JO1605) (ATGGGATGCATTCGAGTAAATACGC) and an antisense oligonucleotide containing half an *Eco*RV site (underlined) (JO2684) (atcTGGAACTCGAGCGTTTTG). The PCR product was cloned into the unique *Eco*RV site of a modified pREP3x vector containing GFP.

### Assay of β-galactosidase Activity

Assays were performed using a method modified from Dohlman *et al.* (1995) [45], [54]. *S. pombe* cells were cultured to a density of ∼5x10^6^ cells/ml in DMM and 500µl aliquots transferred to 2 ml Safe-Lock tubes (Eppendorf, Hamburg, Germany) containing 5 µl of the appropriate ligand (in HPLC-grade methanol). Tubes were incubated at 29°C for 16 h on a rotating wheel, and 50 µl transferred to 750 µl Z-buffer containing 2.25 mM *ο*-nitrophenyl-β-D-galactopyranoside (ONPG). Reactions were stopped after 90 min by adding 200 µl of 2 M Na_2_CO_3_ and β-galactosidase activity calculated as optical density at 420 nm (OD_420_) per 10^6^ cells (determined using a Coulter Channelyzer).

### Confocal Microscopy

In order to measure cell size at division and percentage of multi-septated cells, strains were grown in low nitrogen containing DMM medium to an ∼5x10^6^ cells/ml and stained with calcofluor white (Sigma), which specifically stains cell wall and septum [54], [55]. Minimums of 200 cells, chosen at random, were used in calculating septation rate for each mutant. For life cell imaging cells were placed on a solid DMM (2% agarose) pad on a CoverWell™ imaging chamber (Grace Bio-Labs, Oregon, USA) coverslip was placed over the cells on the agar pad and sealed with a Vaseline, Lanolin and Paraffin equal parts by weight mixture to prevent drying of the sample. Images were then obtained using a True Confocal Scanner Leica TCS SP5 microscope (Leica Microsystems Ltd., Milton Keynes, UK). Series of images were taken using Z stacks at time  = 0 h and subsequently every 15 min for a period of 16 h. The imaging procedure was set up such that cells were focused at 0 h and the Z position noted (Z_focus_). The Z-stack was then defined such that 20 Z-slice images would be obtained from Z_focus_ - 10 µm to Z_focus_ +10 µm to allow for drift in the focal plane over the course of the experiment. The time-series experiment was setup such that both bright field and fluorescence images were generated, and imported into ImageJ (http://rsb.info.nih.gov/ij/). Following analysis the image in the z–stack with the highest intensity was chosen.

### Flow Cytometry

Flow cytometry was performed using a Becton, Dickinson and Company (BD) LSR II flow cytometer (BD Biosciences, Oxford, UK). For cell cycle progression analysis, cells were grown under standard conditions and following sonication, harvested by centrifugation and fixed in 1 ml of ice cold 70% ethanol overnight. 300 µl of fixed cells were then washed in 3 ml of fresh 50 mM sodium citrate and re-suspended in 500 µl of fresh 50 mM sodium citrate containing 0.1 mg/ml RNase A. Cells were incubated at 37°C for 2 h before the addition of 500 µl of 50 mM sodium citrate containing 8 g/ml propidium iodide (Sigma). Up to 30,000 particles per sample were then analyzed using the flow cytometer, measuring the intensities of staining with propidium iodide. Excitation was achieved using a 488 nm laser, and emission detected using a 575/26 nm band pass filter with a 550 nm long pass filter. All analysis was performed using FACSDiva v4.1 (BD Biosciences).

#### Data analysis

Data were analyzed using linear and non-linear regression as appropriate using GraphPad Prism v6.0b (GraphPad Software Inc, San Diego, CA). Statistical significance was determined using a one-way ANOVA with a Tukey multiple comparison post-test or an un-paired Student’s t test.

## Supporting Information

Figure S1 **Characterization of Gnr1p a potential Gβ-subunit mimic in the pheromone-response pathway.** Pheromone-dependent transcription for cells either lacking or overexpressing (using the thiamine repressible nmt1 promoter) Gnr1p, was determined using the sxa2>lacZ reporter. Cells were stimulated with pheromone for 16 h in minimal media and assayed for β-galactosidase production using ONPG. Activity is expressed as OD_420_ units per 10^6^ cells (see methods).
(TIF)

Figure S2 **The N-terminal domain alone of Gpa1 is sufficient to ensure correct plasma membrane localization.** The strains JY546 (h^−^, cyr1^−^, sxa2>lacZ), JY1314 (h^−^, cyr1^−^, sxa2>lacZ, gnr1^−^) and JY1628 (h^−^, cyr1^−^, sxa2>lacZ, cpc2^−^) containing pGFP or p^1−40^Gpa1-GFP were imaged using fluorescence microscopy. Scale bars 10 µm. The N-terminal 40 amino acids of Gpa1 are sufficient to promote plasma membrane localization of GFP. This suggests that Gpa1p does not have a requirement for a classical Gβγ to enable plasma membrane localization.
(TIF)

Figure S3 Pheromone-dependent transcription for the strains JY546 (h^−^, cyr1^−^, sxa2>lacZ), JY1662 (h^−^, cyr1^−^, sxa2>lacZ, cpc2^−^, +oe-RACK1^+^) and JY1663 (h^−^, cyr1^−^, sxa2>lacZ, +oe-RACK1^+^) was determined using the sxa2>lacZ reporter (A). Mammalian RACK1 was expressed using the thiamine repressible nmt1 promoter and cells were cultured in the absence of thiamine to ensure maximal levels of transcription. Cells were stimulated with pheromone for 16 h in minimal media and assayed for β-galactosidase production using ONPG. Activity is expressed as OD_420_ units per 10^6^ cells (see methods). (B) The strains JY546 and JY1663 were treated described in Figure 3, and the number of cells containing a 1C content of DNA (expressed as a percentage of total cells) determined. Consistent with overexpression of Cpc2, RACK1 containing cells fail to desensitize from pheromone stimulation and remain arrested for the time frame analyzed.
(TIF)
