# Expression of Concern: The Role of the RACK1 Ortholog Cpc2p in Modulating Pheromone-Induced Cell Cycle Arrest in Fission Yeast

**DOI:** 10.1371/journal.pone.0225013

**Published:** 2019-11-25

**Authors:** The PLOS ONE Editors

After this article [1] was published, concerns were raised about the following calcofluor white staining results reported in Figs 1, 2 and 5:

- In the 8h, 16h, and 32h panels for JY546(*cyr1*^*-*^, *sxa2*^*-*^) in Fig 2A, and the 8h panel for JY1628(*cyr1*^*-*^, *sxa2*^*-*^, *cpc2*^*-*^) in Fig 2B, there appear to be discontinuities in the background image when levels are adjusted.
- In the JY1578(*cyr1*^*-*^, *sxa2*^*-*^, *oe-cpc2*^*+*^) panels shown in Fig 2C, when levels are adjusted it appears that contrast levels differ between the cells and the background in the 0 hour, 8 hour and 16 hour panels. There is also a rectangle in the 0h panel and an area where background appears discontinuous with surrounding area in the 8h panel.
- In Fig 5B, the image for JY948(*cyr1*^*-*^, *sxa2*^*-*^, *pmp1*^*-*^) appears to have different contrast levels between the cells and surrounding background.
- The image for strain JY1716(*sxa2*^*-*^, *pmp1*^*-*^) in Fig 5B shows a strong similarity to the 16h panel in Fig 2C for strain JY1711(*sxa2*^*-*^,*oe-cpc2*^*+*^*)*.
- The image for strain JY710(*sxa2*^*-*^, *pyp2*^*-*^) in Fig 5D shows a strong similarity to the panel in Fig 1C for strain JY1711(*sxa2*^*-*^,*oe-cpc2*^*+*^).

**Fig 2:**
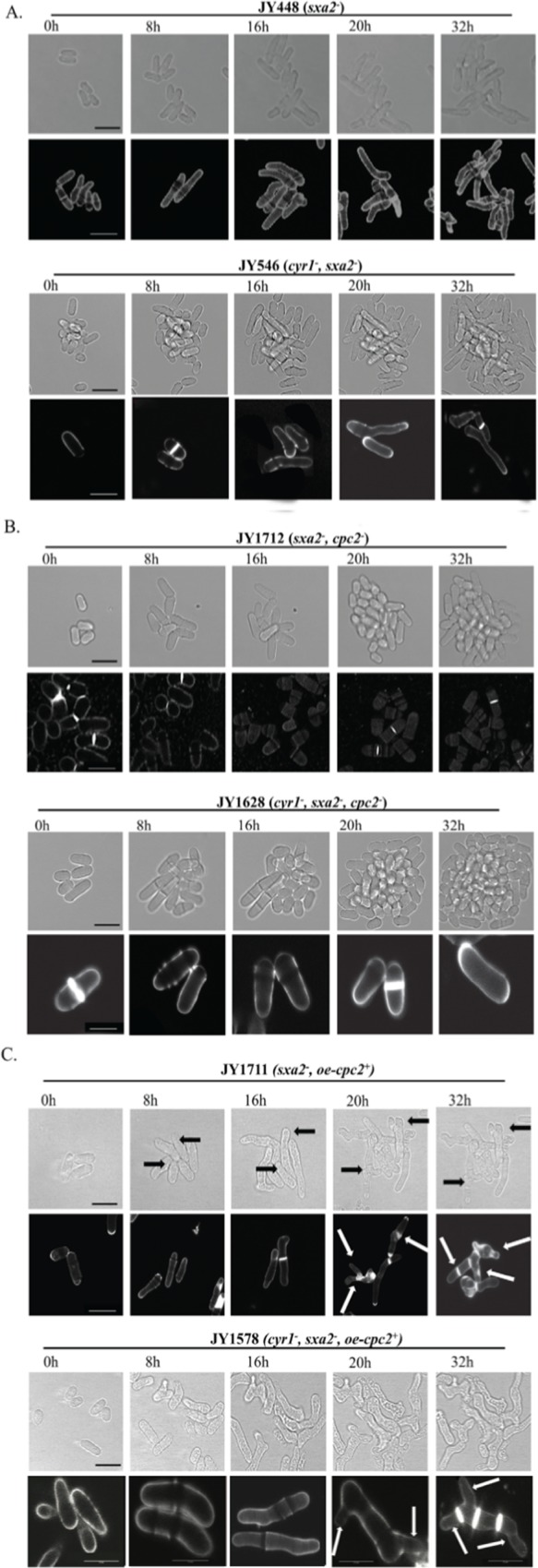
Cpc2p has profound morphological effects upon pheromone-stimulated cells. The strains (A) JY448 (h^−^, sxa2^−^) and JY546 (h^−^, cyr1^−^, sxa2>lacZ); (B) JY1712 (h^−^, sxa2^−^, cpc2^−^) and JY1628 (h^−^, cyr1^−^, sxa2>lacZ, cpc2^−^); (C) JY1711 (h^−^, sxa2^−^+oe-cpc2^+^), JY1578 (h^−^, cyr1^−^, sxa2>lacZ+oe-cpc2^+^), were grown to mid-exponential phase over 32 h in minimal media. Cells were then imaged using bright field microscopy on pads containing 10 μM of pheromone (see methods). Cells were also stained with calcofluor white (lower panels A-C) to visualize septation. Scale bars 10 μm. Prolonged exposure to pheromone for cells overexpressing Cpc2p (oe-cpc2^+^) results in multiple projection tips and a failure to undergo cytokinesis. Cells lacking Cpc2p fail to generate the classical shmoo formation as observed for control cells.

**Fig 5:**
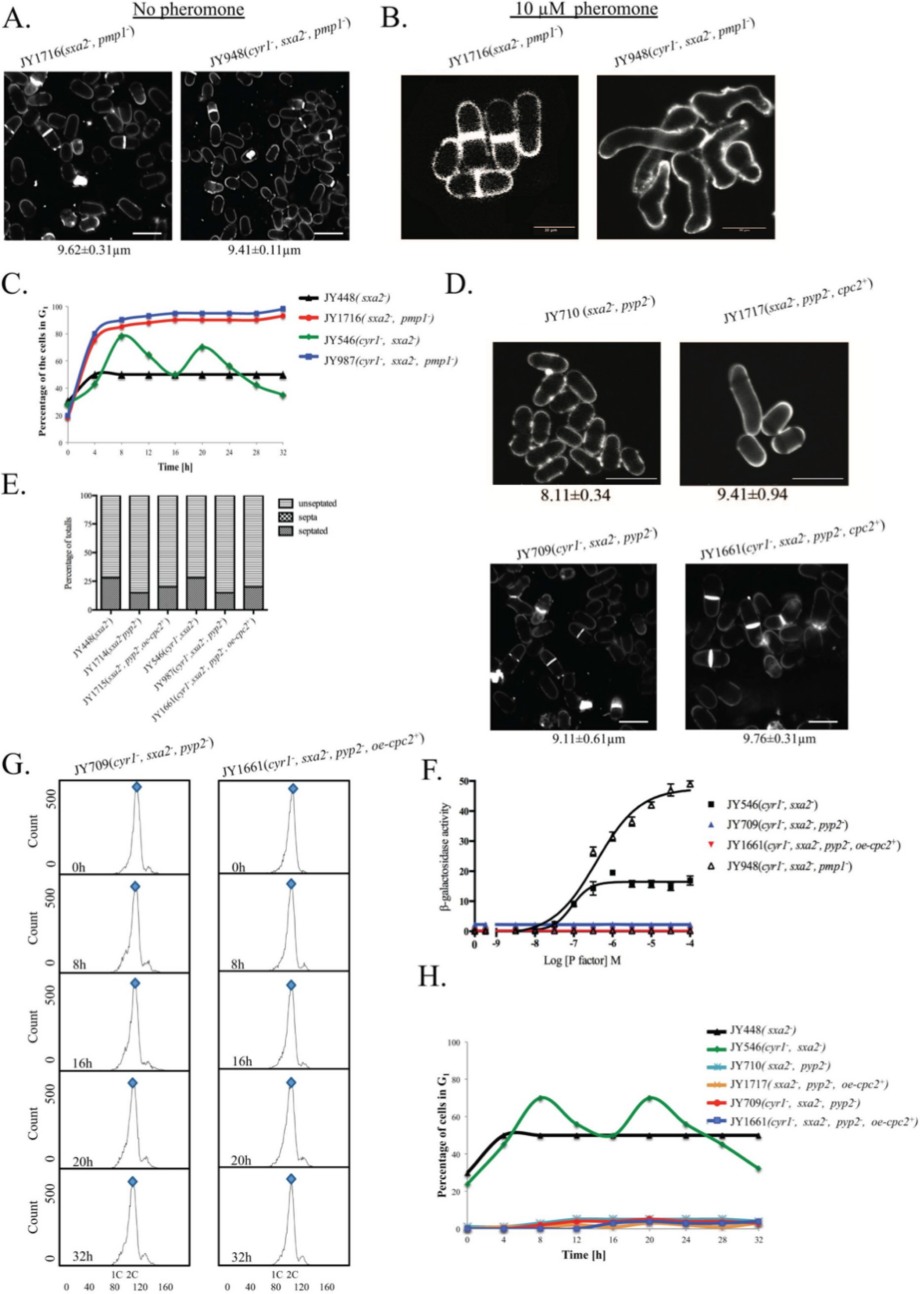
Overexpression of Cpc2 in pheromone stimulated cells mimics prolonged pheromone stimulation. (A) Cell morphology and size, at division (micrometers ± S.D.) for the strains JY1716 (h^−^, sxa2^−^, pmp1^−^) and JY948 (h^−^, cyr1^−^, sxa2>lacZ, pmp1^−^) grown in minimal medium at 29°C and stained with calcofluor white. (B) Strains from A strains were exposure to 10 μM of pheromone for 32 h and stained with calcofluor white. (C) The percentage of cells containing a 1C content (arrested in G_1_) for the strains JY448, JY1716, JY546 and JY948 as determined using flow cytometry. Cells lacking Pmp1p show a failure to exit from a G1 arrest analogous to strains where the cpc2 ORF has been deleted. (D) Cell morphology and size, at division (micrometers ± S.D.) for strains JY710 (h^−^, sxa2^−^ pyp2^−^) and JY1717 (h^−^, sxa2^−^, pyp2^−^+oe-cpc2^+^) grown in minimal medium at 29°C and stained with calcofluor white (top panel). Cell morphology and size at division (micrometers ± S.D.) for the strains JY709 (h^−^, cyr1^−^, sxa2>lacZ, pyp2^−^) and JY1661 (h^−^, cyr1^−^, sxa2>lacZ, pyp2^−^+oe-cpc2^+^) grown in minimal medium at 29°C and stained with calcofluor white (bottom panel). (E) Numbers of non-septated, septated and multiple septa containing cells for the strains JY448, JY1714, JY1715, JY546, JY987 and JY1661 were determined from 400 individual cells. Values shown correspond to the percentages of the total population. Cells were stained with calcofluor white, to enable visualization of septum material. (F) Pheromone-dependent transcription for the strains JY546, JY709, JY1661 and JY948 was determined using the sxa2>lacZ reporter. Cells were stimulated with pheromone for 16 h in minimal media and assayed for β-galactosidase production using ONPG. Activity is expressed as OD_420_ units per 10^6^ cells. Values are means of triplicate determinations ± S.E.M. (G) The strains JY709 and JY1661 were grown in minimal medium containing 10 μM of pheromone for the times indicated. Cells were harvested and fixed prior to staining with propidium iodide prior to analysis using flow cytometry (see methods). The proportion of cells exhibiting 1C or 2C DNA content was determined using FACSDiva v4.1 software for the assigned gates indicated by the blue and red shapes. (H) The percentage of cells containing a 1C content (arrested in G_1_) as determined for the strains JY448, JY546, JY710, JY1717, JY709 and JY1661.

The authors are unable to clarify the concerns raised about background irregularities in Fig 2A–2C but acknowledge the apparent discontinuities. The authors noted that in preparing Fig 2C the cell images were mounted on black backgrounds and a cell which was out of focus in the 0h panel was covered with an overlying rectangle. According to the authors, except for this instance, no cells were obscured or cropped out of the images. In the updated figure, the panels in question in Fig 2C have been replaced with alternative images from the original experiments; Fig 2A and 2B are unchanged. The underlying raw image for Fig 2A, JY546(*cyr1*^*-*^, *sxa2*^*-*^), 16h panel is provided in S1 File.

The authors apologize for the Fig 5 issues and noted that they were due to figure preparation errors. They commented that the image of JY948(*cyr1*^*-*^, *sxa2*^*-*^, *pmp1*^*-*^) cells in Fig 5B was placed on a black background in preparing the figure, but that the cell image accurately reflects data from the original slide. The incorrect data were included in Fig 5B, JY1716(*sxa2*^*-*^, *pmp1*^*-*^) panel, and in Fig 5D, JY710(*sxa2*^*-*^, *pyp2*^*-*^) and JY1717(*sxa2*^*-*^, *pyp2*^*-*^, *cpc2*^*+*^) panels. In the updated Fig 5, these panels are replaced with the correct images from the original experiments. The underlying raw image for the original Fig 5B, 1716(*sxa2*^*-*^, *pmp1*^*-*^) panel is included in S2 File.

Original data are no longer available for the other figure panels discussed above and other results reported in the article. Due to the nature and extent of image issues and lack of underlying image data for the figures in question, the *PLOS ONE* Editors issue this Expression of Concern.

Some of the above concerns were raised in 2014. The *PLOS ONE* Editors apologize for our delay in resolving this matter and thank the authors for replying promptly to journal queries.

## Supporting information

S1 File Underlying image for Fig 2A, JY546(*cyr1*^*-*^, *sxa2*^*-*^), 16h panel. (PDF)

S2 File Underlying image for Fig 5B, 1716(*sxa2*^*-*^, *pmp1*^*-*^) panel. (PDF)
