# Supplementary figures and images for: The Role of the RACK1 Ortholog Cpc2p in Modulating Pheromone-Induced Cell Cycle Arrest in Fission Yeast
Source: PLoS One. 2013 Jul 3;8(7):e65927. doi: 10.1371/journal.pone.0065927 (PMC3701009; doi:10.1371/journal.pone.0065927)

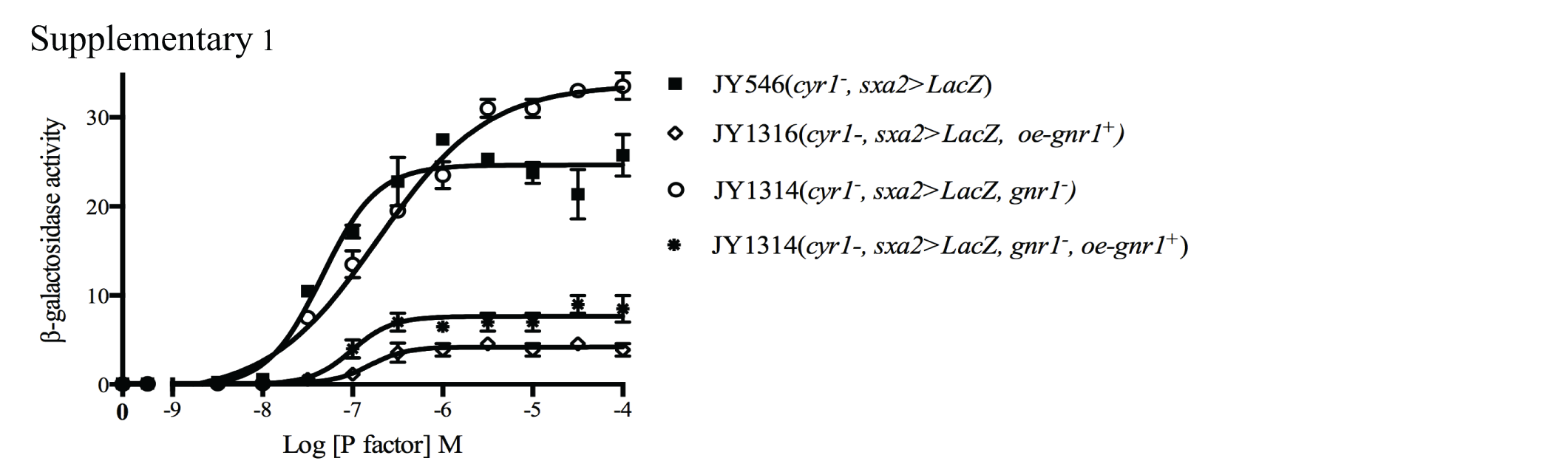

Supplement: Figure S1 — Characterization of Gnr1p a potential Gβ-subunit mimic in the pheromone-response pathway. Pheromone-dependent transcription for cells either lacking or overexpressing (using the thiamine repressible nmt1 promoter) Gnr1p, was determined using the sxa2>lacZ reporter. Cells were stimulated with pheromone for 16 h in minimal media and assayed for β-galactosidase production using ONPG. Activity is expressed as OD420 units per 106 cells (see methods). (TIF) [file pone.0065927.s001.tif]

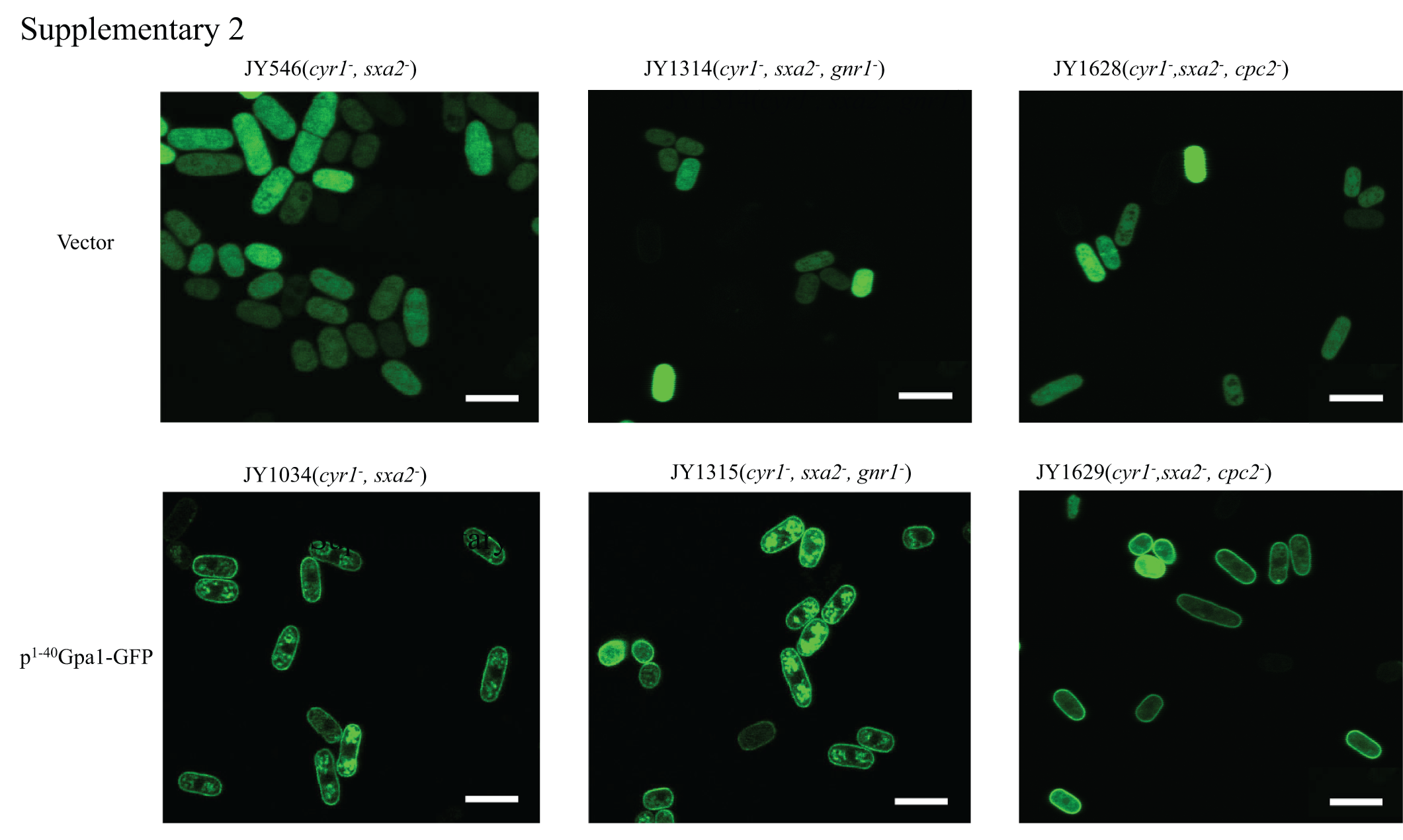

Supplement: Figure S2 — The N-terminal domain alone of Gpa1 is sufficient to ensure correct plasma membrane localization. The strains JY546 (h−, cyr1−, sxa2>lacZ), JY1314 (h−, cyr1−, sxa2>lacZ, gnr1−) and JY1628 (h−, cyr1−, sxa2>lacZ, cpc2−) containing pGFP or p1−40Gpa1-GFP were imaged using fluorescence microscopy. Scale bars 10 µm. The N-terminal 40 amino acids of Gpa1 are sufficient to promote plasma membrane localization of GFP. This suggests that Gpa1p does not have a requirement for a classical Gβγ to enable plasma membrane localization. (TIF) [file pone.0065927.s002.tif]

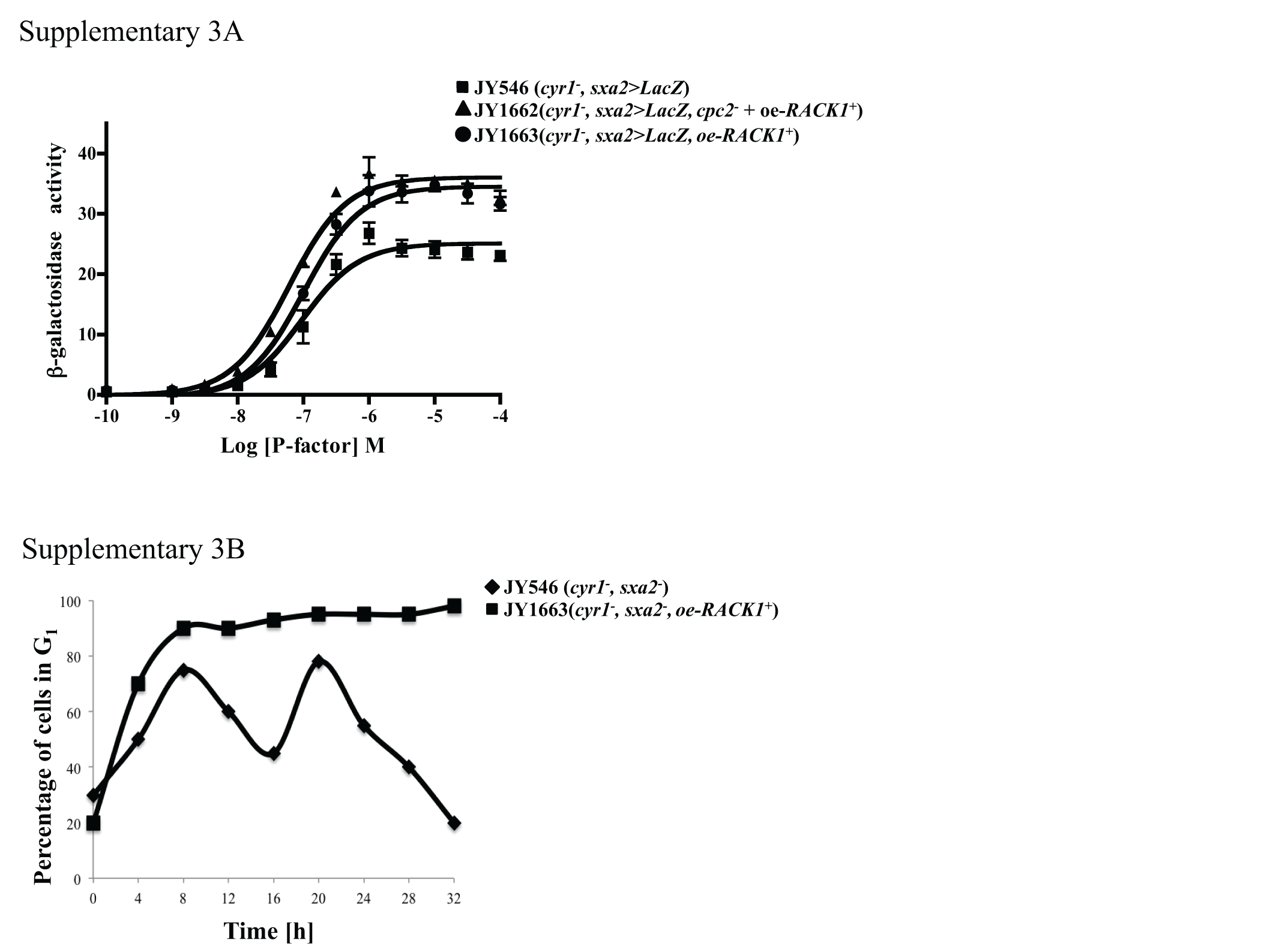

Supplement: Figure S3 — Pheromone-dependent transcription for the strains JY546 (h−, cyr1−, sxa2>lacZ), JY1662 (h−, cyr1−, sxa2>lacZ, cpc2−, +oe-RACK1+) and JY1663 (h−, cyr1−, sxa2>lacZ, +oe-RACK1+) was determined using the sxa2>lacZ reporter (A). Mammalian RACK1 was expressed using the thiamine repressible nmt1 promoter and cells were cultured in the absence of thiamine to ensure maximal levels of transcription. Cells were stimulated with pheromone for 16 h in minimal media and assayed for β-galactosidase production using ONPG. Activity is expressed as OD420 units per 106 cells (see methods). (B) The strains JY546 and JY1663 were treated described in Figure 3, and the number of cells containing a 1C content of DNA (expressed as a percentage of total cells) determined. Consistent with overexpression of Cpc2, RACK1 containing cells fail to desensitize from pheromone stimulation and remain arrested for the time frame analyzed. (TIF) [file pone.0065927.s003.tif]
