# Supplementary figures and images for: Expression of Concern: The Role of the RACK1 Ortholog Cpc2p in Modulating Pheromone-Induced Cell Cycle Arrest in Fission Yeast
Source: PLoS One. 2019 Nov 25;14(11):e0225013. doi: 10.1371/journal.pone.0225013 (PMC6876880; doi:10.1371/journal.pone.0225013)

0 100 200

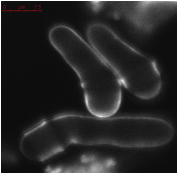

Supplement: S1 File — (PDF) [file pone.0225013.s001.pdf]

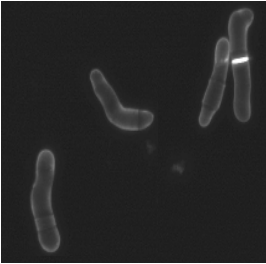

Supplement: S2 File — (PDF) [file pone.0225013.s002.pdf]
